# The Influence of Reactive PS-PVD Process Parameters on the Microstructure and Thermal Properties of Yb_2_Zr_2_O_7_ Thermal Barrier Coating

**DOI:** 10.3390/ma15041594

**Published:** 2022-02-21

**Authors:** Paweł Pędrak, Marek Góral, Kamil Dychton, Marcin Drajewicz, Malgorzata Wierzbinska, Tadeusz Kubaszek

**Affiliations:** Research and Development Laboratory for Aerospace Materials, Rzeszow University of Technology, Powstańców Warszawy 12, 35-959 Rzeszów, Poland; pedrak@prz.edu.pl (P.P.); kdychton@prz.edu.pl (K.D.); drajewic@prz.edu.pl (M.D.); mwierzb@prz.edu.pl (M.W.); tkubaszek@prz.edu.pl (T.K.)

**Keywords:** reactive PS-PVD, PS-PVD, ytterbium zirconate, TBC, plasma spray

## Abstract

Ytterbium zirconate (Yb_2_Zr_2_O_7_) is one of the most promising materials for yttria-stabilized zirconia (YSZ) replacement as a thermal barrier coating (TBCs) application. In the presented report, the experimental synthesis of Yb_2_Zr_2_O_7_ coating using novel Reactive Plasma Spray Physical Vapor Deposition (Reactive PS-PVD) is described. The obtained coating, irrespective of the power current (1800, 2000 and 2200 A), was characterized by a hybrid structure and a thickness of about 80–110 μm. The results of XRD phase analysis showed the formation of ytterbium zirconate in the coating but the presence of ytterbium and zirconium oxides was also detected. The oxides were not observed in calcinated powder. The decrease in thermal conductivity with power current increase was observed. It was the result of higher thickness and better columnar structure of the coating obtained using higher power current of the plasma torch.

## 1. Introduction

From many years, thermal barrier coatings (TBCs) have been widely used for protection of gas turbine hot section parts against high temperature [1]. For increasing temperature, different types of ceramic materials have been considered as an alternative to yttria-stabilized zirconia oxide: mulite, rare-earth oxides, alumina oxide and silicates [2]. Pyrochlores are one of the most promising materials used for TBC application [3]. The different method of thermal spray processes [4] as well as EB-PVD might be used for their formation [5]. They are characterized by lower thermal conductivity in comparison with conventional YSZ (yttria-stabilized zirconia) [6]. Moskal et al. [7] investigated the gadolinium zirconate. Sun et al. [8] developed the technology of Sm_2_Zr_2_O_7_ ceramic coating production using the plasma-spraying process. Moskal et al. [9] proposed the two concepts of ceramic coatings formation: single-layer Nd_2_Zr_2_O_7_ as well as a double layer coating (DLC) with a YSZ and Nd_2_Zr_2_O_7_ outer layer. This same concept of Gadolinum Zirconate double-layer coating was proposed by Mahade et al. [10] to increase the thermal fatigue of TBCs. Wang et al. [11] incorporated the new suspension plasma spraying (SPS) process for production of lanthanum zirconate. This process was also developed for gadolinium zirconate production [12]. Multicomponent pyrochlores such as (Sm_0.2_La_0.8_)_2_(Zr_0.7_Ce_0.3_)_2_O_7_ [13] and La_2_(Zr_0.75_Ce_0.25_)_2_O_7_ [14] for use for TBC production were also investigated. The other concept is co-doping of gadolinium zirconate by Yb_2_O_3_ and Sc_2_O_3_ [15]. The other type of pyrochlore developed is europium zirconate [16]. Recently, the new concepts of pyrochlores have been considered: cerate and hafnate of europium [17] and samarium [18]. The other idea is to develop high-entropy zirconates [19,20]. The selected properties of pyrochlores, for example resistance to hot corrosion, were investigated by Moskal et al. [21,22]. There are not many references regarding ytterbium zirconate. The hot corrosion in the presence of V_2_O_5_ was investigated by Li [23]. An investigation conducted by He et al. [24] showed that Yb_2_Zr_2_O_7_ might by synthetized from pure ZrO_2_ and Yb_2_O_3_ powders using the laser excitation process. The obtained results showed the presence of pure oxides in the obtained coating. Ytterbia-stabilized zirconia might be also used for doping of gadolinium zirconate used for TBCs [25]. Ren et al. [26,27] investigated the multicomponent LaYbZr_2_O_7_ as a plasma sprayed coating as a TBC application. One of promising technologies for production of advanced ceramic coatings is the Plasma Spray Physical Vapor Deposition (PS-PVD) process [28,29]. The Gd_2_Zr_2_O_7_ is the only type of pyrochlore used for production of ceramic coatings by the PS-PVD method [30]. The different types of powder preparation methods and their influence on the columnar structure of the coating was investigated. Comparative study of the properties of double-layer TBCs containing gadolinium zirconate and yttria-stabilized zirconia (YSZ) with a single-layer Gd_2_Zr_2_O_7_ coating was also performed [31]. In our previous research, we proposed a new Reactive PS-PVD method and the formation of well-investigated gadolinium zirconate during reaction of gadolinia and zirconia oxides in a plasma plume [32]. In present article, we synthetized Ytterbia Zirconate using a novel reactive PS-PVD process.

## 2. Materials and Methods

The Inconel 713C-type nickel superalloy was used as a base material. The NiC°CrAlY bond coat was formed by a low pressure plasma spraying method (LPPS) using Amdry 386 powder (Oerlikon-Metco, Switzerland) containing (wt.%): Co–23%; Cr–17%; Al–12%; Y–0.45%; Ni–bal. The used process parameters were based on our previous research [33]: power current 1600 A, plasma gasses flow Ar: 35 NLPM (normal liters per minute), He: 60 NLPM, powder feed rate: 1 g/min, process chamber pressure: 40 mbar, spray distance 0.95 m.

The pure zirconium (ZrO_2_) and ytterbium (Yb_2_O_3_) oxides were used as a feedstock material for powder production. They were mixed in the following proportion (ratio: Yb_2_O_3_ 65.6 wt.%., ZrO_2_ 34.4% wt.%) for synthesis of ytterbium zirconate. For comparison, the prepared powder mixture was calcinated at 1500 °C. Powders were mixed with polyvinyl alcohol additive (about 2 wt.%) and spray dried. For synthesis of ytterbium zirconate during reactive PS-PVD process, the methodology of coating formation proposed by He [24] was used. The ytterbium zirconate coating was formed in the reactive PS-PVD process during the following reaction:

Yb_2_O_3_ + 2ZrO_2_ → Yb_2_Zr_2_O_7_ (1)

Both the bond-coat and ceramic topcoat were produced using the LPPS-Hybrid system (Oerlikon-Metco, Switzerland) in the Research and Development Laboratory for Aerospace Materials at Rzeszow University of Technology, Poland. The basic process parameters of the topcoat production were selected based on our previous research [32,33,34,35,36]. The experimental process parameters are presented in Table 1.

The microstructure of the obtained samples and the thickness were examined using a scanning electron microscope (S-3400N, Hitachi, Japan). Phase composition analysis was conducted for all obtained ceramic coatings as well as for those calcinated at 1500 °C powder for comparison. It was conducted using an X-ray diffractometer ARL X’TRA (CuKα radiation Bragg–Brentano geometry value of the angle 20–90, Thermo Scientific Corporation, Waltham, MA, USA). For identification of the phase components, the ICDD-PDF4-2019 crystallographic database was used.

Specific heat measurement of the ytterbium zirconate powder was conducted using the STA 449 F3 Jupiter cpDSC device (Netzsch, Serb, Germany) by the differential calorimetry method according to ASTM E 1269 and DIN 51 007 standards. The specific heat value was measured in the temperature range 700 to 1100 °C. The used heating speed was 10 °C/min. The powder for specific heat measurement was prepared by firing graphite with ytterbium zirconate coating deposited using 2200 A power current at 1000 °C for 12 h and ground in a ball mill.

The thermal diffusivity measurement was performed using a LFA 427 device (Netzsch, Germany) in the temperature 700 to 1100 °C (Ar flow 50 mL/min). Before thermal diffusivity measurement, samples were covered by a graphite layer. The Cape–Lehman method and the two-layer model of the coating and were applied for analysis. The substrate with a metallic bond coat was adopted as the 1st layer, while the 2nd layer (top coat) was Yb_2_Zr_2_O_7_ formed during the reactive PS-PVD processes.

## 3. Results and Discussion

### 3.1. The Microstructure 

**Figure 1:**
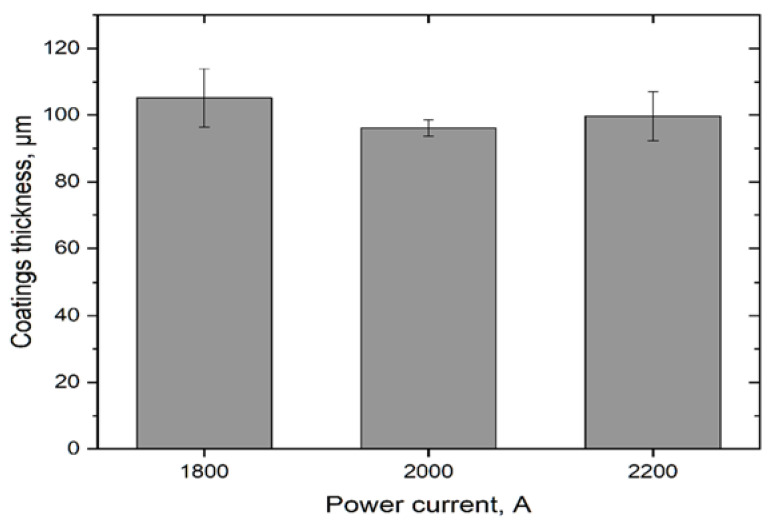
The average thickness of ytterbium zirconate TBCs formed in the reactive PS-PVD process using different power currents (1800, 2000, 2200 A).

**Figure 2:**
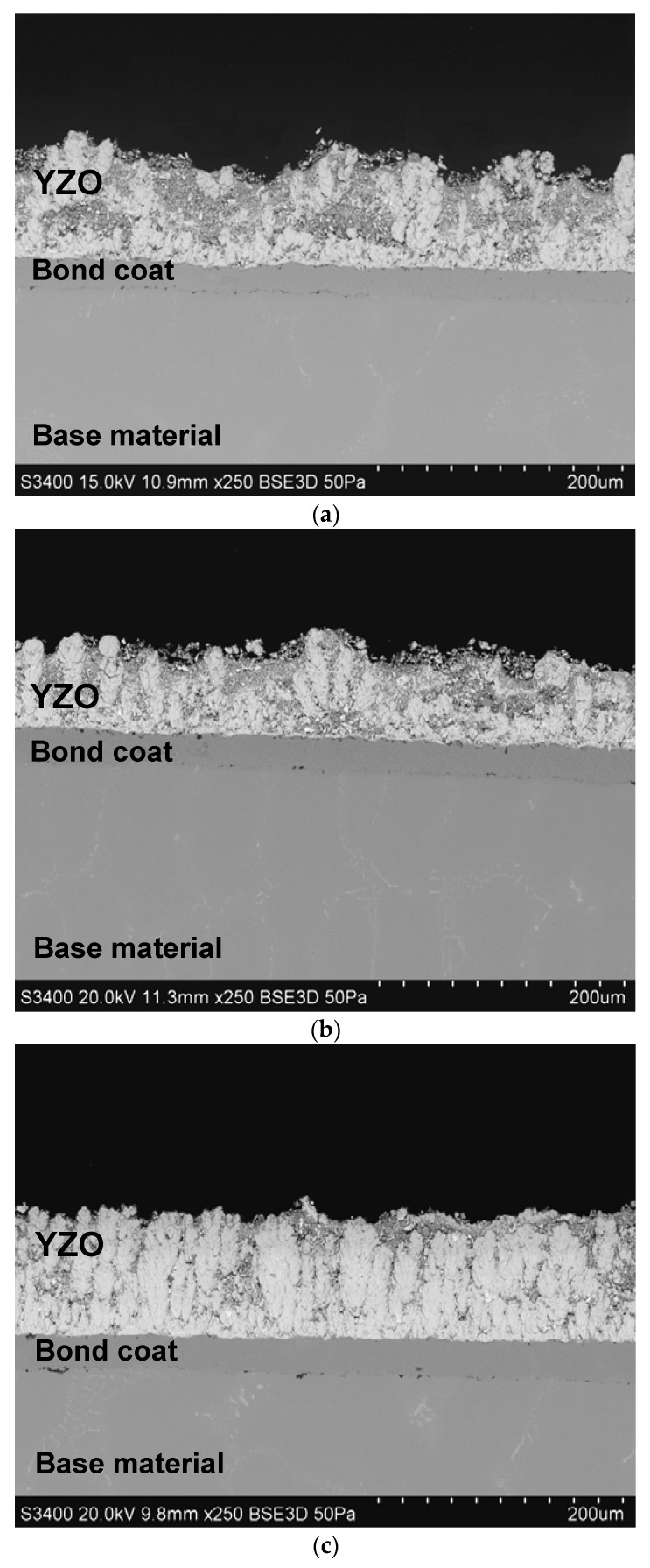
Microstructures of Yb_2_Zr_2_O_7_ (GZO) layers made with different power current values: (**a**) YZO-1800 A, (**b**) YZO-2000 A, (**c**) YZO-2200 A.

The deposited coatings were characterized by a mixed columnar and dense structure according to the PS-PVD coatings model growth proposed by Zhang [36]. The thickness of the obtained coatings was in the range 80–100 μm (Figure 1). The discontinuous zone with a columnar structure was formed when the lower power current (1800, 2000 A) was used (Figure 2a,b). This model structure might be described as a hybrid—columns and spheroidal grains were observed [37]. When the power current was increased to 2200 A, the continuous quasi-columnar structure was formed [38] but small spheroidal particles were still observed (Figure 2c). They formed as a result of secondary crystallization of material observed when longer spray distance of PS-PVD processes was used [39]. The obtained results of microstructure analysis showed that structure of YZO coating are similar to previously synthetized gadolinium zirconate coating [32]. 

### 3.2. Phase Composition

**Figure 3:**
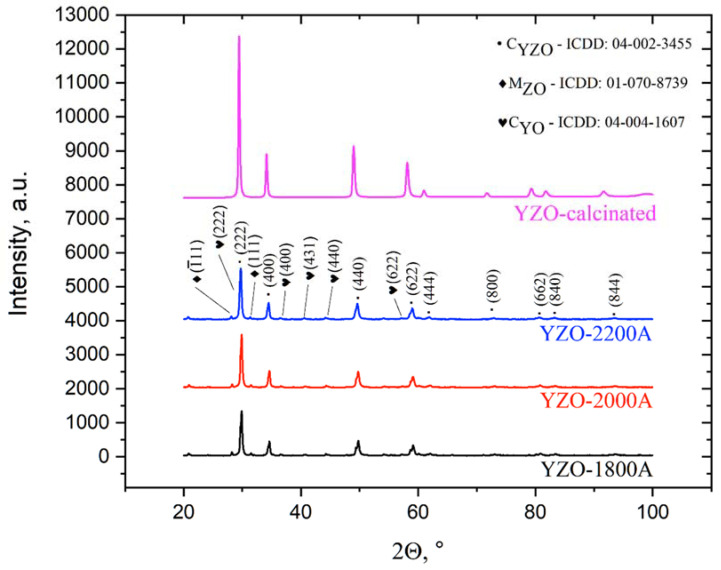
XRD patterns of the Yb_2_Zr_2_O_7_ coating deposited in the reactive PS-PVD process using different power currents I = 1800, 2000, and 2200 A) and calcinated at 1500 °C mixture of ZrO_2_ and Yb_2_O_3_ powders (YZO-calcinated).

The XRD phase analysis showed, in the synthetized coating, the presence of three phases: ytterbium zirconate (YZO, ICDD card no. 04-002-3455), monoclinic zirconia oxide (MZO ICDD card no. 01-070-8739) and cubic ytterbium oxide (CYO, ICDD card no. 04-004-1607) (Figure 2, YZO-1800, YZO-2000, YZO-2200). There were not differences of phase composition in the coating formed using different power currents. The obtained results suggest that ytterbium zirconate was the main component of the obtained coating. On the other hand, the presence of ytterbium and zirconium oxides indicated that not all mixed particles formed Yb_2_Zr_2_O_7_. The both oxides were not detected in powder calcinated at 1500 °C (YZO-calcinated, Figure 3).

### 3.3. Thermal Properties

**Figure 4:**
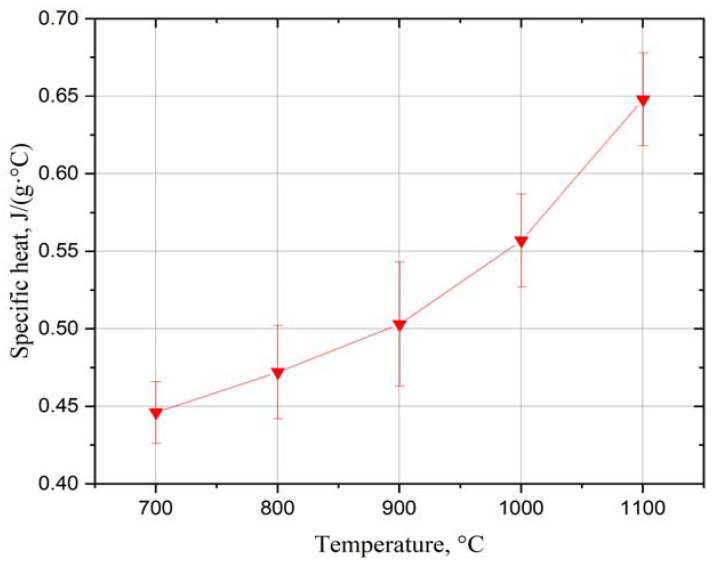
Specific heat of the Yb_2_Zr_2_O_7_ coating in temperature range 700–1100 °C.

**Figure 5:**
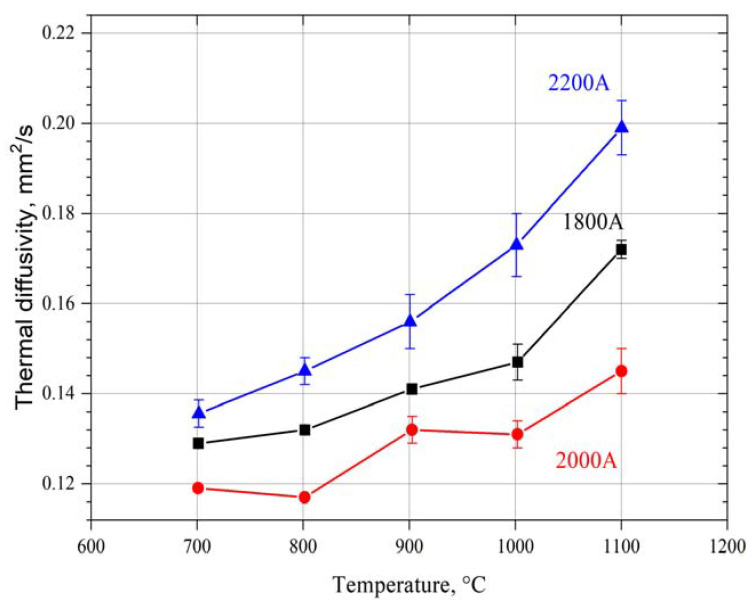
Thermal diffusivity of synthetized Yb_2_Zr_2_O_7_ in the reactive PS-PVD process using different power currents in the temperature range 700–1100 °C.

**Figure 6:**
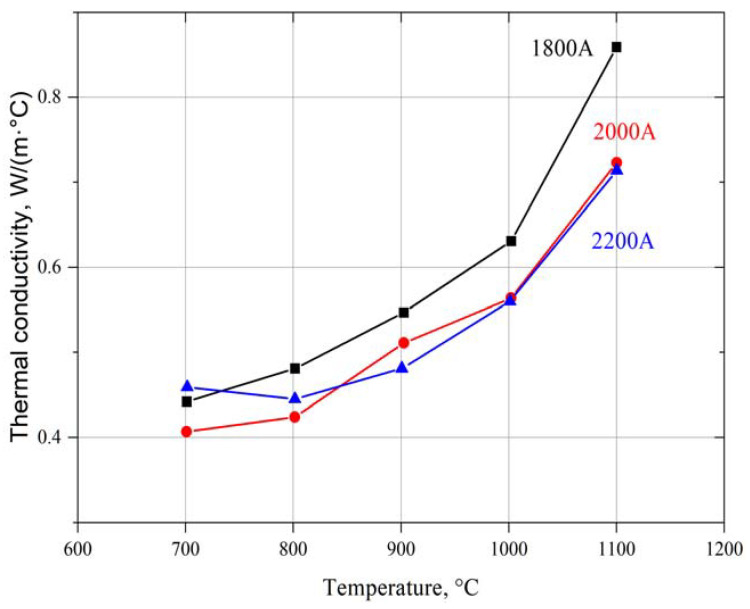
Thermal conductivity of synthetized Yb_2_Zr_2_O_7_ in the reactive PS-PVD process using different power currents in the temperature range 700–1100 °C.

Results of the specific heat (Figure 4) and thermal diffusivity (Figure 5) measurement were used for thermal conductivity calculation (Figure 6), while keeping the material density constant. However, in the conductivity calculations, the presence of pores was not taken into account due to the difficulty of determining their geometry and type.

The thermal diffusivity of the ytterbium zirconate coating rises with increasing temperature from 0.11–0.14 mm^2^/s at 700 °C to 0.14–0.2 at 1100 °C (Figure 5). The highest thermal diffusivity was measured for the coating obtained using the power current 2200 A.

The results of thermal conductivity of the synthetized coating (Figure 6) were much lower in comparison with yttria-stabilized zirconia oxide obtained using the PS-PVD process [40]. A similar value was measured for previously developed Gd_2_Zr_2_O_7_ coatings [34,40]. The thermal conductivity rises with temperature (700–1100 °C) from about 0.4 to almost 0.8 W/(m°C). The highest thermal conductivity value was measured for the coating, which was produced using the lowest power current (YZO-1800 A), probably according to its dense structure. When the power current was increased up to 2000 or 2200, the thermal conductivity decreased. The observed trends in thermal conductivity changes with temperature are almost opposite the thermal conductivity of the Yb_2_Zr_2_O_7_ coating synthetized using the laser excitation method [24]. On the other hand, the Reactive PS-PVD forms an YZO coating characterized by other structure (hybrid or quasi-columnar), which has different thermal properties. 

## 4. Conclusions

1. Ytterbium zirconate (Yb_2_Zr_2_O_7_) was successfully formed during a reactive PS-PVD process using only a mixture of pure Yb_2_O_3_ and ZrO_2_ powder.
2. When the lower power current was used (1800 and 2000 A) a hybrid structure for the coating was obtained. The increase in power current to 2200 A caused the quasi-columnar structure to form during the reactive PS-PVD process.
3. The XRD phase analysis showed non-significant differences in the coating using different power current values. The Yb_2_Zr_2_O_7_ was the main component of the obtained coating. On the other hand, the presence of ytterbium and zirconium oxides indicates incomplete transformation of the entire mixture of oxides. The complete reaction was only observed in the mixture of powders calcined at 1500 °C.
4. Higher thermal conductivity was calculated for the Yb_2_Zr_2_O_7_ coating formed using a higher power current (2200 A). It was a result the of quasi-columnar structure of coating characterized by higher thermal conductivity in comparison with the hybrid structure formed using the 1800 A power current.

## Figures and Tables

**Table 1:** Parameters used for production of ytterbium zirconate coatings.

Process Name	Power Current, A	Argon Flow, NLPM	Helium Flow, NLPM	Powder Feed Rate, g/min
YZO-1800A	1800	35	60	2
YZO-2000A	2000	35	60	2
YZO-2200A	2200	35	60	2

## Data Availability

There is no additional data in this place.

## References

[1] Mondal K., Nuñez L., Downey C.M., Van Rooyen I.J. (2021). Thermal barrier coatings overview: Design, manufacturing, and applications in high-temperature industries. Ind. Eng. Chem. Res..

[2] Cao X.Q., Vassen R., Stoever D. (2004). Ceramic materials for thermal barrier coatings. J. Eur. Ceram. Soc..

[3] Vaßen R., Sebold D., Stöver D. (2008). Corrosion behavior of new thermal barrier coatings. Ceram. Eng. Sci. Proc..

[4] Vassen R., Stuke A., Stöver D. (2009). Recent developments in the field of thermal barrier coatings. J. Therm. Spray Technol..

[5] Saruhan B., Schulz U., Bartsch M. (2007). Developments in processing of ceramic top coats of EB-PVD thermal barrier coatings. Key Eng. Mater..

[6] Moskal G., Witala B., Rozmyslowska A. (2009). Thermal diffusivity of RE_2_Zr_2_O_7_—Type ceramic powders intended for TBCs deposited by APS. Arch. Mater. Sci. Eng..

[7] Moskal G., Rozmysłowska A. (2010). Microstructure and thermal diffusivity of Gd_2_Zr_2_O_7_ powders. Adv. Mater. Res..

[8] Sun X., Wang Q., Liu Y., Ning X., Wang C. (2012). Preparation and properties of Sm_2_Zr_2_O_7_ ceramics for thermal barrier coatings by APS. Adv. Mater. Res..

[9] Moskal G., Swadźba L., Hetmańczyk M., Mendala J., Sosnowy P. (2012). Characterisation of the microstructure and thermal properties of Nd_2_Zr_2_O_7_ and Nd_2_Zr_2_O_7_/YSZ thermal barrier coatings. J. Eur. Ceram. Soc..

[10] Mahade S., Curry N., Björklund S., Markocsan N., Nylén P. (2016). Failure analysis of Gd_2_Zr_2_O_7_/YSZ multi-layered thermal barrier coatings subjected to thermal cyclic fatigue. J. Alloy. Compd..

[11] Wang C., Wang Y., Wang L., Hao G., Sun X., Shan F., Zou Z. (2014). Nanocomposite Lanthanum Zirconate Thermal Barrier Coating Deposited by Suspension Plasma Spray Process. J. Therm. Spray Technol..

[12] Mahade S., Curry N., Björklund S., Markoscan N., Nylén P., Vaßen R. (2017). Functional performance of Gd_2_Zr_2_O_7_/YSZ multi-layered thermal barrier coatings deposited by suspension plasma spray. Surf. Coat. Technol..

[13] Xu Z.H., Shen Z.Y., Mu R.D., He L.M. (2018). Phase structure, thermophysical properties and thermal cycling behavior of novel (Sm_0.2_La_0.8_)_2_(Zr_0.7_Ce_0.3_)_2_O_7_ thermal barrier coatings. Vacuum.

[14] Yang P., An Y., Zhao D., Li Y., Zhou H., Chen J. (2020). Structure evolution, thermal properties and sintering resistance of promising thermal barrier coating material La_2_(Zr_0.75_Ce_0.25_)_2_O_7_. Ceram. Int..

[15] Guo Y., He W., Guo H. (2020). Thermo-physical and mechanical properties of Yb_2_O_3_ and Sc_2_O_3_ co-doped Gd_2_Zr_2_O_7_ ceramics. Ceram. Int..

[16] Mikuskiewicz M., Moskal G. (2019). Solid state synthesis of europium zirconate based material. Arch. Metall. Mater..

[17] Mikuśkiewicz M., Moskal G., Migas D., Stopyra M. (2019). Thermal diffusivity characterization of europium zirconate, cerate and hafnate. Ceram. Int..

[18] Stopyra M., Moskal G., Niemiec D. (2015). Synthesis and thermal properties of europium zirconate and hafnate via solid state reaction and polymerized complex method. Surf. Coat. Technol..

[19] Li F., Zhou L., Liu J.-X., Liang Y., Zhang G.-J. (2019). High-entropy pyrochlores with low thermal conductivity for thermal barrier coating materials. J. Adv. Ceram..

[20] Cong L., Zhang S., Gu S., Li W. (2021). Thermophysical properties of a novel high entropy hafnate ceramic. J. Mater. Sci. Technol..

[21] Moskal G., Jucha S., Mikuśkiewicz M., Migas D., Jasik A. (2020). Atypical decomposition processes of Sm_2_Zr_2_O_7_ + 8YSZ dual-phase TBCs during hot corrosion. Corros. Sci..

[22] Jasik A., Moskal G., Jucha S., Kuligowski K. (2019). Surface condition of La_2_Zr_2_O_7_ based tbc system after hot corrosion in molten sulfate Na_2_SO_4_ salts. Ochr. Przed Koroz..

[23] Li S., Liu Z.-G., Ouyang J.-H. (2010). Hot corrosion behaviour of Yb_2_Zr_2_O_7_ ceramic coated with V_2_O_5_ at temperatures of 600–800 °C in air. Corros. Sci..

[24] He J., Huang Y., Hu M., Jiang M., Qiao Y., Li S., Zeng H. (2020). Thermal barrier materials of Yb_2_O_3_-ZrO_2_ system synthesized by laser excitation. Mater. Res. Express.

[25] Zhang Y., Guo L., Wang C., Ye F. (2015). Toughening effect of Y_b_2O_3_ stabilized ZrO_2_ doped in Gd_2_Zr_2_O_7_ ceramic for thermal barrier coatings. Mater. Sci. Eng. A.

[26] Ren X., Zhao M., Wan C., Zheng Y., Pan W. (2015). High-Temperature Aging of Plasma Sprayed Quasi-Eutectoid LaYbZr_2_O_7_-Part I: Phase Evolution. J. Am. Ceram. Soc..

[27] Ren X., Zhao M., Wan C., Zheng Y., Pan W. (2015). High Temperature Aging of Plasma Sprayed Quasi-Eutectoid LaYbZr_2_O_7_-Part II: Microstructure & Thermal Conductivity. J. Am. Ceram. Soc..

[28] Mauer G., Jarligo M.O., Rezanka S., Hospach A., Vaßen R. (2015). Novel opportunities for thermal spray by PS-PVD. Surf. Coat. Technol..

[29] Góral M., Swadźba R., Kubaszek T. (2020). TEM investigations of TGO formation during cyclic oxidation in two- and three-layered Thermal Barrier Coatings produced using LPPS, CVD and PS-PVD methods. Surf. Coat. Technol..

[30] Li S., He W., Shi J., Wei L., He J., Guo H. (2020). PS-PVD gadolinium zirconate thermal barrier coatings with columnar micro-structure sprayed from sintered powder feedstocks. Surf. Coat. Technol..

[31] Zhu R.-B., Zou J.-P., Mao J., Deng C.-M., Liu M. (2021). A comparison between novel Gd_2_Zr_2_O_7_ and Gd_2_Zr_2_O_7_/YSZ thermal barrier coatings fabricated by plasma spray-physical vapor deposition. Rare Met..

[32] Pedrak M., Dychton K., Drajewicz M., Goral M. (2021). Synthesis of Gd_2_Zr_2_O_7_ coatings in novel Reactive PS-PVD process. Coatings.

[33] Goral M., Kotowski S., Dychton K., Drajewicz M., Kubaszek T. (2014). Influence of low pressure plasma spraying parameters on MCrAlY bond coat and its microstructure. Key Eng. Mater..

[34] Goral M., Kotowski S., Nowotnik A., Pytel M., Drajewicz M., Sieniawski J. (2013). PS-PVD deposition of thermal barrier coatings. Surf. Coat. Technol..

[35] Góral M., Pytel M., Kubaszek T., Drajewicz M., Simka W., Nieuzyła L. (2021). The new concept of thermal barrier coatings with Pt + Pd/Zr/Hf-modified aluminide bond coat and ceramic layer formed by PS-PVD method. High Temp. Mater. Processes.

[36] Zhang X., Zhou K., Liu M., Deng C., Deng C. (2019). Deposition mechanism of 7YSZ thermal barrier coatings prepared by plasma spray-physical vapor deposition. Sci. Sin. Technol..

[37] Gao L., Wei L., Guo H., Gong S., Xu H. (2016). Deposition mechanisms of yttria-stabilized zirconia coatings during plasma spray physical vapor deposition. Ceram. Int..

[38] Zhang B., Wei L., Gao L., Guo H., Xhu H. (2017). Microstructural characterization of PS-PVD ceramic thermal barrier coatings with quasi-columnar structures. Surf. Coat. Technol..

[39] Zhang X.F., Zhou K.S., Deng C.M., Liu M., Deng Z.Q., Deng C.G., Song J.B. (2016). Gas-deposition mechanisms of 7YSZ coating based on plasma spray-physical vapor deposition. J. Eur. Ceram. Soc..

[40] Qiu S.-Y., Shi J., Li S., Ma Y., Guo H.-B. (2021). Model on thermal conductivity prediction of quasi-columnar structured coating by plasma spray physical vapor deposition. Ceram. Int..

